# Impact of Pitching on Infraspinatus Muscle Elasticity in High School Baseball Pitchers: A Continuous Shear Wave Elastography Study

**DOI:** 10.3390/diagnostics15060749

**Published:** 2025-03-17

**Authors:** Hitoshi Shitara, Ren Koda, Tsuyoshi Tajika, Tsuyoshi Sasaki, Masataka Kamiyama, Ryosuke Miyamoto, Yuhei Hatori, Koichiro Yanai, Kurumi Nakase, Fukuhisa Ino, Takuma Kachi, Yoshiki Yamakoshi, Hirotaka Chikuda

**Affiliations:** 1 Department of Orthopaedic Surgery, Gunma University Graduate School of Medicine, 3-39-15 Showa-machi, Maebashi 371-8511, Japan; 2 Graduate School of Science and Technology, Gunma University, 1-5-1 Tenjin-cho, Kiryu-shi 376-8515, Japan

**Keywords:** infraspinatus, elasticity, high school, continuous shear wave elastography (C-SWE), baseball

## Abstract

**Background/Objectives**: The repetitive overhead throwing of baseball stresses the posterior shoulder, including the rotator cuff and capsule, causing stiffness, tissue thickening, and dysfunction. Previous studies on collegiate baseball players have linked these changes to glenohumeral internal rotation deficits, pain, and injuries. However, these studies primarily used acoustic radiation force impulse-based shear wave elastography (SWE), which has limitations, including tissue heating and lack of portability. The acute effects of pitching on infraspinatus (ISP) muscle elasticity in high school pitchers remain unclear. Therefore, this study aimed to evaluate the acute impact of pitching on ISP muscle elasticity in high school baseball pitchers using continuous SWE (C-SWE), which is a safer and more portable method. The relationship between ISP muscle elasticity and pitching load was also examined. **Methods**: ISP muscle shear wave velocity (SWV), shoulder range of motion, and strength were evaluated in high school baseball pitchers. The participants were categorized into pitching and non-pitching groups based on whether they pitched with full effort on the day of their medical checkup. C-SWE was used to assess ISP muscle elasticity. **Results**: The pitching group had considerably higher ISP muscle SWV on the dominant side than the non-pitching group (*p* = 0.008). A significant positive correlation was observed between pitch and ISP muscle SWV (r = 0.467, *p* = 0.003). **Conclusions**: Repetitive pitching acutely increases ISP muscle stiffness in high school pitchers, contributing to posterior shoulder tightness. C-SWE is a safe and practical method for assessing tissue elasticity and developing injury prevention strategies.

## 1. Introduction

The posterior rotator cuff and capsule are subjected to substantial distraction forces during the follow-through phase of baseball throwing, leading to a marked increase in tissue stiffness [1,2,3,4,5]. Repetitive posterior microtrauma and subsequent tissue healing are believed to cause the thickening of the posterior shoulder capsule [6,7,8], whereas stiffness of the muscular components is associated with their response to strain [9]. Increased stiffness in the posterior capsule and muscles has been linked to glenohumeral internal rotation deficits (GIRD) in the throwing arm, as well as shoulder pain, injuries, and fatigue [10,11,12,13,14].

College baseball players with >15° reduction in horizontal adduction (HA) and internal rotation (IR) of the throwing shoulder demonstrate significantly increased stiffness in the infraspinatus (ISP) and lower trapezius muscles compared to their counterparts with <15° reduction in HA and IR of the throwing shoulder [15]. Furthermore, the throwing motion imposes substantial strain on the posterior shoulder musculature, as demonstrated by the increased elasticity of the ISP muscle immediately after pitching, which persists for up to 24 h [16]. However, these studies focused only on college-level baseball players. In previous studies [15,16], muscle elasticity has been evaluated using ultrasonic shear wave elastography (SWE) with acoustic radiation force impulse (ARFI) imaging. However, this method has notable limitations, including the risk of tissue heating near the bones due to intense ultrasonic waves [17,18,19]. Additionally, detecting shear waves requires specialized high-frame-rate ultrasound devices, adding to system complexity and costs. Moreover, the size and lack of portability also make these devices impractical for use in sports settings and on the field.

**Figure 1:**
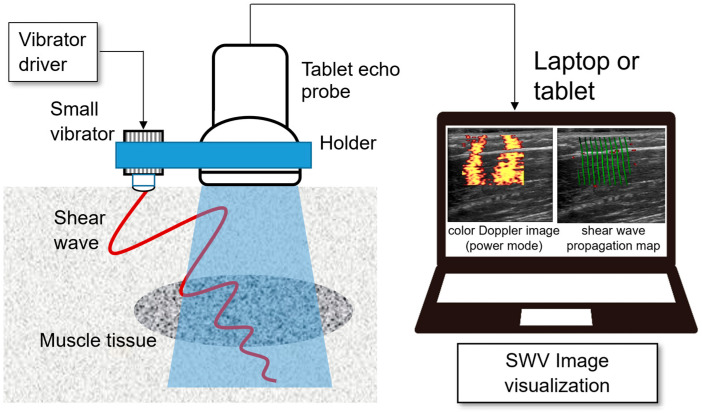
Schematic diagram of C-SWE. Shear waves are produced by a small vibrator placed on the body’s surface, while an ultrasound probe detects their propagation. In C-SWE, a complex orthogonal detection signal (IQ signal) detected orthogonally with respect to the received signal is obtained by the tablet echo device, which is connected to the laptop. The acquired IQ signal is signal-processed and reproduced by the shear wavefront in real-time. SWV, shear wave velocity; C-SWE, continuous shear wave elastography.

To overcome these limitations, researchers have explored alternative approaches, including mechanical excitation, to estimate shear wave velocity (SWV) [20,21,22]. Among these, continuous SWE (C-SWE), developed by Yamakoshi et al., represents a significant advancement [23,24,25]. C-SWE uses a compact vibrator to generate and visualize shear waves, thereby enabling tissue stiffness assessment (Figure 1).

One of the advantages of C-SWE is that it is an easy-to-apply method for small, portable ultrasound diagnostic equipment, as the phase of the shear wave can be obtained without the need to modify conventional ultrasound equipment. C-SWE can be implemented using a combination of a handheld ultrasound probe and a laptop or tablet device for video display, providing a cost-effective and versatile method for quantitatively assessing tissue stiffness.

In contrast to ARFI-based methods, C-SWE can be integrated into various ultrasound systems, including portable tablet-based devices, making it suitable for diverse settings such as medical examination rooms, bedside evaluations, physical therapy sessions, and sports. By utilizing mechanical excitation rather than acoustic radiation, C-SWE ensures the real-time observation of shear wave propagation with enhanced safety for living tissues. This innovative approach offers a simple yet effective solution for assessing tissue elasticity with minimal additional equipment.

To the best of our knowledge, no study has specifically addressed the acute impact of pitching on ISP muscle elasticity in high school pitchers. Therefore, this study aimed to examine the influence of pitching on ISP muscle elasticity in high school baseball pitchers using C-SWE, which is a safer and more advanced method. We also examined the relationship between changes in ISP muscle elasticity and pitching load, including the number of full pitches thrown. This will contribute to a better understanding of how repetitive pitching influences posterior shoulder function and tissue properties in younger athletes.

## 2. Materials and Methods

### 2.1. Participants

This cross-sectional study adhered to the Strengthening the Reporting of Observational Studies in Epidemiology guidelines and included high school baseball pitchers who underwent medical checkups in 2024. All participants were males and belonged to organized baseball teams. In accordance with previous studies [26,27], the inclusion criteria required that each pitcher (1) actively participated in pre-season workouts and (2) had no restrictions on their pitching or throwing activities. The exclusion criteria included (1) a history of injury (e.g., fracture) to the throwing arm or (2) inability to throw or restricted pitching activity due to a shoulder or elbow condition. All participants provided written informed consent before enrollment in this study. Its protocol was approved by the Institutional Review Board of Gunma University Hospital (approval number: 1003).

### 2.2. Medical Checkups

As described in previous studies [27], the shoulder and elbow condition of participants was assessed during pre-season medical checkups. To prevent confirmation bias, the examiners were unaware of the dominant side of the participants. The evaluations included measurements of age, height, weight, body mass index (BMI), baseball experience, shoulder range of motion (ROM), shoulder muscle strength, and ISP muscle elasticity. The number of full pitches thrown on the day of the medical checkup was recorded since a previous study demonstrated that pitching significantly increased ISP muscle elasticity within 24 h [16].

### 2.3. Shoulder and Elbow ROM

A certified orthopedic surgeon (Y.M.) used a digital protractor to measure the participants’ passive ROM in the dominant and non-dominant shoulders, including abduction external rotation (ER), abduction IR, and HA, according to the standardized method described in previous studies [27,28,29,30,31]. The reliability of this measurement technique has been validated in previous research, demonstrating a high intra-rater and inter-rater reliability [26].

The measurements were performed with the participants in a supine position. To measure passive ER and IR, the examiner stabilized the scapula by applying a posterior force to the coracoid process while another certified orthopedic surgeon placed a digital protractor on the forearm. In contrast, the examiner stabilized the scapula’s axillary border to measure passive HA, while another certified orthopedic surgeon placed a digital protractor on the humerus.

### 2.4. Shoulder Strength

A certified orthopedic surgeon (Y.M.) used a PowerTrack II Commander handheld dynamometer (J-Tech Medical, Salt Lake City, UT, USA) to measure the strength of the bilateral prone ER (PER) and prone IR (PIR) [27]. Shoulder muscle strength was measured with the participants in the prone position. The participants were instructed to abduct their humerus and flex their elbow to 90°. Subsequently, the examiner stabilized the humerus and placed the arm in a neutral position. The participants rotated their arms externally or internally with maximum effort against the dynamometer. For PER, the dynamometer was placed on the dorsal forearm, 5 cm proximal to the wrist extension crease. The PIR was placed on the volar forearm 5 cm proximal to the wrist flexion crease. Each measurement was repeated three times and recorded. Median data values were analyzed, and the ratio of dominant-arm PER to dominant-arm PIR strength was calculated for each participant.

### 2.5. Assessment of SWV in the ISP Muscle

The elasticity of the ISP muscle was measured as SWV. The validity and reliability of this approach have been previously established [32]. SWV was measured in the bilateral ISP muscles using a tablet echo device (Finggal Link Co., Ltd., Iwate, Japan) [33], and a custom-made vibrator (frequency range: 60–150 Hz, vibration amplitude: ≤1 mm) was applied to produce continuous shear waves. To ensure a stable distance between the ultrasonic transducer and vibrator, we used a specially designed elastic attachment, which was created with a 3D printer (Anycubic Kobra 2 Neo, Shenzhen Anycubic Technology Co., Ltd., Shenzhen, China). The tablet-based ultrasound device was connected to a laptop PC, where quadrature detection was applied to the received signal to extract a complex quadrature detection (IQ) signal. Signals from 64 elements of the linear probe were collected in 16 data packets and stored as text files for further analysis. The shear wave frequency was fixed at 78.1 Hz, aligning with the pulse repetition frequency of the ultrasound system divided by 16. An orthopedic surgeon with over 15 years of musculoskeletal ultrasound expertise performed all the measurements.

**Figure 2:**
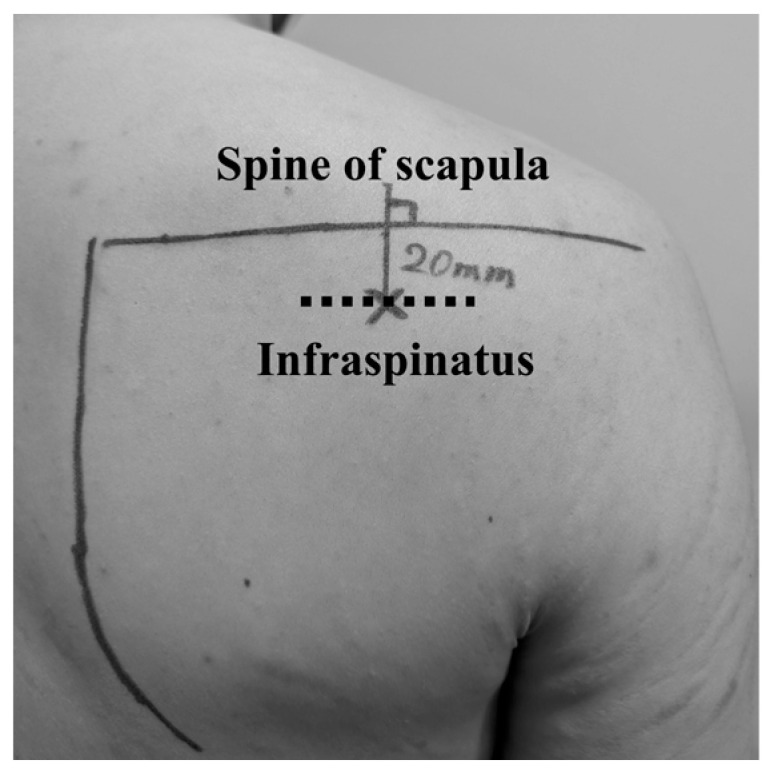
Measured points of ultrasound examinations.

Participants were positioned in a seated posture with their throwing arms resting at their sides. To assess the ISP muscle, an ultrasound transducer was positioned 2 cm below the midpoint of the scapular spine [3,15,16] (Figure 2).

Prior to the assessment, the transducer and excitation point were carefully adjusted to align shear wave propagation with the muscle fiber direction, which was verified using shear-wave wavefront images. Once the appropriate propagation pattern was confirmed, continuous measurements were recorded. The most uniform wavefronts that met the predefined analysis conditions were automatically selected for determining the propagation speed of shear waves. SWV analysis was conducted using dedicated software that extracts SWV from the IQ signals obtained by the tablet echo device [33]. The power Doppler mode was set to a frame rate of 7.4 fps and an ultrasound frequency of 7.5 MHz. Additionally, the region of interest was defined as 18.6 mm in depth and 30.1 mm in the horizontal direction. Shear wave propagation was visualized at a rate of approximately 2 s per frame [33].

### 2.6. Statistical Analyses

An a priori sample size calculation was conducted using G*Power (version 3.1.9.7; Heinrich Heine University, Düsseldorf, Germany) to determine the required sample size. The effect size (d = 1.065) was based on a previous study [15] that examined ISP muscle elasticity using SWE. A sample size of 40 participants was considered sufficient to detect this effect size with 90% power and an alpha level of 0.05.

The study population was classified into pitching and non-pitching groups according to whether they pitched at their full strength on the day of the medical checkup.

For continuous variables, data are expressed as mean ± standard error of the mean (SEM), and between-group comparisons were made using the Mann–Whitney U test. Categorical variables are presented as frequencies and percentages, with between-group comparisons conducted using the chi-square or Fisher’s exact test, depending on sample size constraints. The association between SWV and clinical parameters was examined using Spearman’s rank correlation coefficient.

IBM SPSS Statistics for Windows, version 29.0 (IBM Japan, Tokyo, Japan) was used for statistical analysis, with significance determined at *p* < 0.05.

## 3. Results

Nine pitchers were in the pitching group and 29 in the non-pitching group. The mean number of full pitches on the day of the medical checkups in the pitching group was 47.2 (SEM: 10.9). No significant differences were found between the two groups in dominant side, age, height, weight, BMI, or baseball experience.

The ISP muscle SWV on the dominant side was significantly higher in the pitching group than in the non-pitching group (4.4 ± 0.4 m/s vs. 3.4 ± 0.1 m/s, *p* = 0.008). However, significant differences were found in the ISP muscle SWV on the non-dominant side between the groups.

**Figure 3:**
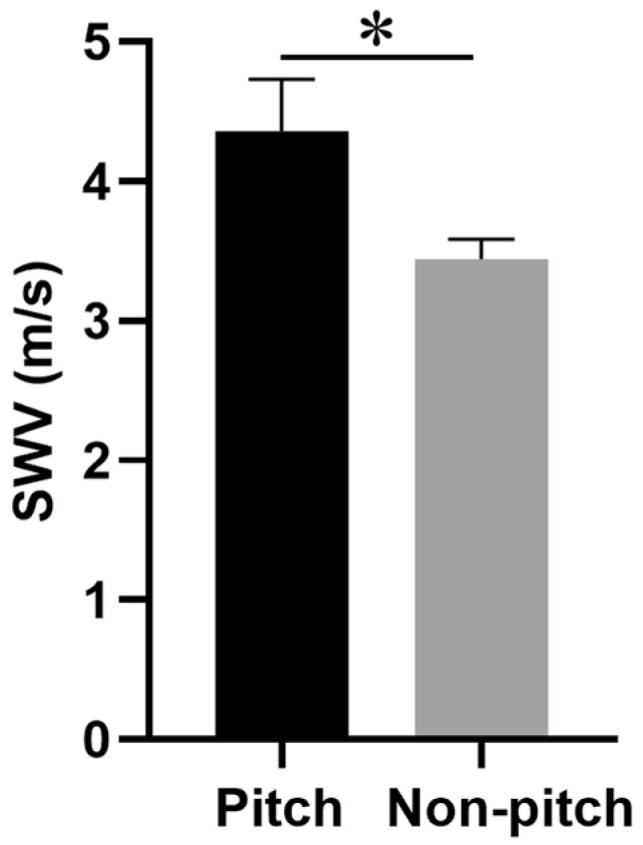
ISP muscle SWV on the dominant side between the pitching and non-pitching groups. * *p* < 0.05. Bars: mean; error bars: standard of the mean. SWV, share wave velocity; ISP, infraspinatus.

Furthermore, the IR on both sides was significantly lower in the pitching group than in the non-pitching group (dominant side: 38.8 ± 3.6° vs. 56.6 ± 3.0°, *p* = 0.004; non-dominant side: 53.1 ± 4.7° vs. 62.3 ± 1.9°, *p* = 0.036) (Figure 3). No significant differences were found in ER, HA, or shoulder strength of the PER and PIR on either side between the two groups (Table 1).

### Association Between the Number of Full Pitches and ISP Muscle SWV

**Figure 4:**
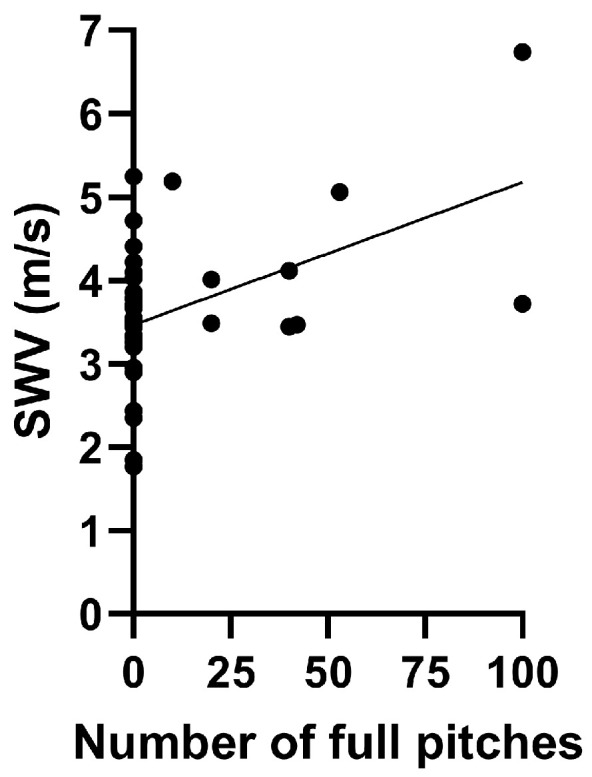
Association between the number of full pitches and ISP muscle SWV on the dominant side. ISP, infraspinatus; SWV, share wave velocity.

We found a significant positive association between the number of full pitches and ISP muscle SWV on the dominant side (r = 0.467, *p* = 0.003) (Figure 4). However, no significant association was found between the number of pitches and ISP muscle SWV on the non-dominant side (r = −0.257, *p* = 0.120).

## 4. Discussion

This study used C-SWE, a safer and more advanced method, to demonstrate that the SWV of the ISP muscle on the dominant side was significantly higher in the pitching group than in the non-pitching group. Furthermore, a significant positive association was found between the number of full pitches and SWV of the ISP muscle on the dominant side. To the best of our knowledge, this is the first study to document these changes in high school baseball pitchers. These findings suggest that repetitive pitching places a high demand on the ISP muscle, leading to an increase in stiffness or elasticity.

### 4.1. Impact on the Elasticity of ISP Muscle/Tendon by Throwing/Pitching

Previous studies reported that repetitive throwing significantly increases the stiffness of the posterior shoulder muscles, including the ISP. Yamaura et al. showed that ISP muscle elasticity in baseball players with a mean age of 26.6 years (range, 24–33 years), as measured using SWE, increased immediately after 100 pitches and remained elevated at 24 h post-throwing, indicating persistent mechanical stress due to repetitive pitching [16]. Similarly, Itoigawa et al. reported increased stiffness in the ISP muscle of the throwing shoulders of college players compared with the non-throwing side [34]. Additionally, they found that increased ISP muscle SWE values and decreased IR abduction were significantly associated with shoulder pain during throwing [34]. Mifune et al. showed increased elasticity of the ISP muscle in college baseball players by comparing the stiff group, which was defined as the throwing side decreasing >15° from the non-throwing side in both the ROMs of HA and IR and the non-stiff group [15]. These results suggest that ISP muscle stiffness is closely linked to posterior shoulder tightness, which may contribute to throwing-related pain and reduced ROM.

This present study extends these findings to high school baseball pitchers by demonstrating the acute impact of pitching on ISP muscle elasticity in a real-world setting. In contrast to previous studies that used an ARFI-based SWE method, which carries potential risks of localized tissue heating near bony structures, this study employed C-SWE. Notably, C-SWE provides a safer alternative with reduced heating effects [35], enabling a more precise assessment of ISP muscle adaptations in younger athletes.

Repetitive pitching places significant mechanical stress on the posterior shoulder, particularly during the follow-through phase, where high eccentric loads are imposed on the ISP muscle to decelerate the arm [1,2]. This eccentric contraction leads to increased passive stiffness as a protective mechanism against excessive strain, potentially contributing to posterior shoulder tightness [36]. Previous studies have suggested that repeated eccentric loading can result in higher collagen deposition and alterations in muscle architecture, such as sarcomere lengthening and increased extracellular matrix stiffness, which may explain the greater ISP muscle stiffness observed in this present study [37,38].

Pitching-induced microtrauma in the ISP muscle may also trigger an inflammatory response, leading to transient muscle stiffness due to edema and localized fibrosis [39]. Yamaura et al. demonstrated that ISP stiffness increased immediately after pitching and remained elevated for 24 h, which could reflect ongoing tissue remodeling [16]. While this may serve as an adaptive mechanism to enhance tissue resilience, prolonged or excessive stiffness could negatively impact shoulder kinematics, increasing the risk of overuse injuries, such as GIRD, superior labrum anterior-posterior tears, and rotator cuff pathology [40,41].

This present study demonstrated a significant positive correlation between the number of full pitches and ISP muscle stiffness, reinforcing the idea that mechanical load directly influences acute changes in muscle elasticity. While increased ISP stiffness may help stabilize the shoulder against excessive strain, monitoring these changes is important to prevent long-term dysfunction. Therefore, future research should investigate how different pitch counts, training regimens, and recovery strategies influence ISP muscle stiffness over time.

### 4.2. Shoulder Functions and Posterior Shoulder Elasticity

A previous study [15] on college baseball players classified those with a >15° reduction in HA and IR in the throwing shoulder as the STIFF+ group, showing significantly greater stiffness in the ISP and lower trapezius muscles than the STIFF- group. This finding highlights the association between increased muscle stiffness and posterior shoulder tightness among baseball players [15].

Lee et al. studied college baseball players and found that posterior shoulder capsule elasticity was significantly greater in the throwing shoulders of players with limited IR (>15° loss) and total rotation (>10° loss) than in those without these limitations [42]. In this group, IR muscle strength was higher, and posterior capsule elasticity showed a significant negative correlation with the ER/IR strength ratio [42]. These findings suggest that repetitive pitching leads to adaptive changes, such as increased stiffness in the ISP and other posterior shoulder structures, which in turn may contribute to reduced shoulder mobility and altered shoulder biomechanics during throwing. In this present study, no significant association was observed between the ISP muscle elasticity and shoulder motion or strength. The discrepancy between the findings of this study and those of previous studies [15,42], such as Mifune et al. [15] and Lee et al. [42], can be attributed to differences in study design, participant characteristics, and grouping criteria. While previous studies grouped participants based on shoulder ROM deficits, this study compared high school pitchers based on their pitching activity. The participants in this study were high school athletes, whereas previous studies focused on college players who likely had greater cumulative exposure to pitching stress, leading to more pronounced changes in the posterior shoulder structures. Younger athletes may also have greater musculoskeletal adaptability, which could mitigate stiffness or structural changes in response to pitching. These factors, combined with potential methodological differences in elastography techniques, may explain the lack of significant associations among ISP muscle elasticity, shoulder motion, and strength in this study. This highlights the need for tailored research designs that consider the developmental and competitive differences among younger athletes.

### 4.3. Acute Changes in Posterior Shoulder Elasticity and Tendon Thickness Following Pitching

Acute quantitative ultrasound studies have documented significant changes in the ISP tendons of young baseball players after 50 pitches, with increased tendon width. These findings suggest a physiological response to pitching stress and its potential role in the development of future pathologies [43]. Similarly, Yamaura et al. [16] demonstrated that pitching significantly increases the stiffness of the posterior shoulder muscles, including the ISP and lower trapezius, immediately after throwing, with stiffness persisting for approximately 24 h. These changes are associated with acute deficits in shoulder ROM, particularly in IR.

Consistent with these prior studies [16,43], this study found a significant positive correlation between the number of pitches and SWV of the ISP muscle on the dominant side. However, contrasting results were reported by Tsurukami et al. [36], who found no significant change in ISP muscle stiffness after 20 pitches, regardless of whether the participants were pitchers or players [36].

The discrepancies between these studies could stem from differences in the intensity and duration of the pitching sessions, the athletic level of the participants, or the timing of post-pitching measurements. For example, the relatively low pitch count (20) in the study by Tsurukami et al. may not have induced sufficient mechanical stress to elicit measurable changes in muscle elasticity. Additionally, variations in the competitive level and physical conditioning of the participants might also influence the degree of shoulder adaptation to pitching stress.

Therefore, future longitudinal studies are warranted to better understand the acute and chronic effects of pitching on ISP muscle elasticity. These studies should explore the relationship between pitch volume and intensity and the timing of measurements to clarify how repetitive pitching affects the posterior shoulder structures and functions over time.

### 4.4. Limitations

This study had some limitations. First, the number of participants in the pitching group was relatively small. However, a power analysis was conducted to determine the minimum required sample size, and the findings indicated that the study was adequately powered to detect changes in ISP muscle elasticity. Future studies with larger sample sizes are warranted to confirm these findings and improve generalizability. Second, we did not evaluate factors that could influence muscle elasticity, such as humeral torsion, the posterior inferior glenohumeral ligament and capsule, and other muscles, including the middle trapezius, lower trapezius, rhomboid, and serratus anterior. Although all participants were assessed in approximately the same shoulder position, variations in humeral torsion may have affected the muscle tension. This is a potential confounder because differences in torsion may influence the relationship between muscle stiffness and rotational ROM. Third, we did not assess pitching biomechanics, which could influence the stress on the posterior shoulder structures. Fourth, this study exclusively included pitchers who underwent medical checkups, and non-pitchers were not part of the study population. Therefore, comparative C-SWE images between pitchers and non-pitchers could not be provided. Two technical considerations should also be noted regarding the use of C-SWE. First, C-SWE requires an external vibrator for shear wave generation in contrast to conventional SWE with ARFI. While this is generally not an issue in MSK imaging, adjustments may be needed when imaging smaller anatomical regions, such as the fingers, where a more compact vibrator may be required. Second, while C-SWE avoids the risk of tissue heating near bones, as observed with ARFI-based SWE, shear waves can reflect off bony structures, leading to standing wave artifacts. To mitigate this, a directional filter was applied in this study to reduce reflected wave interference. However, this approach does not eliminate reflection artifacts, and careful vibrator placement remains essential to improve measurement accuracy.

## 5. Conclusions

This cross-sectional study found that the SWV of the ISP muscle on the dominant side was significantly higher in the pitching group than in the non-pitching group, as measured using C-SWE. Furthermore, a significant positive association was found between the number of full pitches and the SWV of the ISP muscle on the dominant side. These findings suggest that repetitive pitching may lead to increased stiffness in the posterior shoulder muscles, which could be an adaptive response to excessive loads placed on these structures during overhead throwing.

## Figures and Tables

**Table 1:** Baseline characteristics.

	Pitching Group (*n* = 9)		Non-Pitching Group (*n* = 29)		*p*-Value	
Dominant	R 7 (78%) L 2 (22%)		R 22 (76%) L 7 (24%)		1.000	
	Mean	SEM	Mean	SEM	*p*-value	
Age (years)	16.8	0.1	16.4	0.1	0.060	
Height (cm)	176.4	3.1	172.6	1.5	0.233	
Weight (kg)	74.0	4.2	68.0	2.2	0.194	
BMI (kg/m^2^)	23.7	0.9	22.7	0.5	0.360	
Baseball experience (years)	8.2	0.5	9.3	0.3	0.092	
Shoulder elasticity (m/s)						
SWV on the Dom	4.4	0.4	3.4	0.1	0.008	*
SWV on the Ndom	3.3	0.2	3.5	0.1	0.416	
Shoulder ROM (°)						
ER on the Dom	106.6	3.5	103.0	1.8	0.348	
ER on the Ndom	95.7	3.1	99.7	1.6	0.238	
IR on the Dom	38.8	3.6	56.6	3.0	0.004	*
IR on the Ndom	53.1	4.7	62.3	1.9	0.036	*
HA on the Dom	15.8	1.9	17.5	1.0	0.417	
HA on the Ndom	14.9	2.0	16.3	1.2	0.568	
Shoulder strength (kgf)						
PER on the Dom	10.2	1.1	10.3	0.5	0.930	
PER on the Ndom	12.1	1.0	10.4	0.6	0.170	
PIR on the Dom	14.0	1.4	12.4	0.8	0.323	
PIR on the Ndom	13.9	1.2	12.0	0.7	0.203	

* *p* < 0.05. SWV, share wave velocity; ER and IR, ROM in 90° shoulder abduction external and internal rotation, respectively; HA, horizontal adduction; PER, prone external rotation; PIR, prone internal rotation; ROM, range of motion; SEM: standard error of the mean, Dom: Dominant side, Ndom: Non-dominant side.

## Data Availability

Data supporting the findings of this study are available from the corresponding author (H.S.) upon request. The data were not publicly available because they contained information that could compromise the participants’ privacy.

## Abbreviations

The following abbreviations are used in this manuscript:

HA	horizontal adduction
PER	prone external rotation
PIR	prone internal rotation
ROM	range of motion
SEM	standard error of the mean
Dom	dominant side
Ndom	non-dominant side
AFRI	acoustic radiation force impulse
SWE	shear wave elastography
ISP	infraspinatus
C-SWE	continuous shear wave elastography
ER	external rotation
SWV	shear wave velocity
IR	internal rotation
GIRD	glenohumeral internal rotation deficit
BMI	body mass index
